# Applying Landscape Ecology in Local Planning, Some Experiences

**DOI:** 10.3390/ijerph20043410

**Published:** 2023-02-15

**Authors:** Inger-Lill Eikaas, Helene Roussel, Anne-Karine H. Thorén, Wenche E. Dramstad

**Affiliations:** 1 Asplan Viak, 1337 Sandvika, Norway; 2 Nordplan, 6771 Nordfjordeid, Norway; 3 Department of Landscape Architecture and Spatial Planning, The Norwegian University of Life Sciences, P.O. Box 5003 NMBU, 1432 Ås, Norway; 4 Department of Landscape Monitoring, Survey and Statistics Division, P.O. Box 115 NIBIO, 1431 Ås, Norway

**Keywords:** landscape ecology, planning, landscape design

## Abstract

Landscape ecology is repeatedly described as an applied science that can help reduce the negative effects of land-use and land-use changes on biodiversity. However, the extent to which landscape ecology is in fact contributing to planning and design processes is questioned. The aim of this paper is to investigate if and how landscape ecology can be integrated in a planning and design process, and to uncover possible problems that, e.g., landscape architects and planners, may face in such processes. Our conclusion, based on a case study from Asker municipality, Norway, is that such a landscape ecological approach has a lot to offer. However, it is difficult to exploit the potential fully for different reasons, e.g., biodiversity information tends to be specialized, and not easily used by planners and designers, and landscape ecological principles need an adaptation process to be applicable in a real-world situation. We conclude that for the situation to improve, landscape ecologists need to ease this process. In addition, we recommend collaboration across disciplinary boundaries, preferably with a common design concept as a foundation.

## 1. Introduction

The world’s population is expected to reach 9.6 billion people by 2050, an increase of 1.6 billion from the present 8 billion [1]. Combined with climate change and a range of other environmental challenges, an obvious result is an increasing pressure on land resources [2], a pressure also being an important driver of species loss and ecosystem degradation [3]. Furthermore, the land area being a finite resource underlines the need to manage it in a sustainable way [4]. In particular, urban areas are expected to face continued population growth [5]. To accommodate this increased population an increase in built infrastructure is a common approach, often leading to reduced green space [6,7]. However, it is well-documented that urban green infrastructure has numerous positive effects on the urban population [7] and represent habitat to many species [8].

Landscape architects and land-use planners are among the professional practitioners often dedicated to planning land-use change, and therefore may be considered key professions in the quest to ensure the availability of urban green spaces while meeting the challenge outlined by Forman and Wu [9], Where to put the next billion people. As for the role of landscape architects and land-use planners, a relevant question then is whether they have the information and planning tools needed to consider and integrate ecology and the conservation of biodiversity as an aspect of planning land-use.

Landscape ecology is rooted both in geography and ecology. Within landscape ecology, a model has been developed where any part of any landscape can be assessed as representing patches, corridors, or the matrix (the background ecological system), to the species in focus [10]. In brief, the combination of these landscape elements and their configuration into different spatial patterns, are considered important to the ecology of the landscape, e.g., to whether species successfully colonize new areas, to the interaction between species, and ultimately their continued survival [10].

Some authors have pointed to the potential of landscape ecology to contribute to sustainable development [11,12,13]. At the same time, several authors have commented on the lack of practical application of scientific findings in real world changes, e.g., to ensure species conservation or a continued delivery of ecosystem services [14,15,16]. This critique is relevant also to landscape ecology, a science claiming to have a focus on real-world applications [10,11]. Transferring knowledge from theory to practice is difficult, however, as theoretical and general principles need to be ‘translated’ or placed in a spatially explicit context to be applicable in real-world designs [11,14,17]. Furthermore, biologists may not know how their knowledge can be made more useful in a planning context. This is where we believe landscape ecology can be used as a “meeting point” based on the shared concern about spatial patterns, landscape composition, and configuration.

Landscape ecology focuses on landscape content and composition. The agencies that one would suppose were able to apply landscape ecological theory and findings to real-world situations are often public or privately employed professional practitioners, not scientists. Accordingly, landscape ecology has produced several examples of principles and recommendations intended to aid this process [18,19,20,21,22,23,24]. The most common recommendations are to prioritize larger areas, ensure connectivity, minimize distances and contrast where possible, and create heterogeneous environments. However, it is uncertain to which extent the principles have gained acceptance [25,26,27]. This is supported by Van Damme [28], who underlines the need to strengthen the implementation of concepts, principles, and methods of landscape ecology in planning, management, policy, and design. Similarly, Trammell, Carter, Haby, and Taylor [27], p. 2, outlined how “…calls for increased integration are numerous and longstanding”. Additionally, Hersperger, Grădinaru, Pierri Daunt, Imhof, and Fan [26] in their recent extensive review found that while several landscape ecological concepts were frequently used, they conclude that “… landscape ecological concepts have not achieved deep integration into the planning process” (p. 2342).

Several possible explanations have been forwarded to explain what is known as the “science-practice gap” [29], and they can probably be relevant for the topics in focus here. Lack of communication between ecologists and landscape planners is one candidate, lack of knowledge, training, or familiarity may be the other, or maybe ‘ecological interests’ have a lower priority compared to other interests represented in a landscape [11,16], a concern also outlined by Forman and Wu [9]. Additionally, relevant in this context is whether professional practitioners indeed perceive scientific principles and findings as practically applicable. Previous studies have considered a lack of knowledge transfer considered of real use for professional practitioners a main obstacle [15,30,31]. A Norwegian study of planning practices related to biodiversity reported similar results [32]. In this study, landscape ecological principles, such as those developed by Agger [24], were referred to as an important theoretical foundation in their land-use planning. There was also extensive documentation of nature types, habitats, red-listed species, etc. However, the study contained no information about how the municipalities translated the landscape ecological principles to management in the planning processes, making it difficult to conclude regarding the actual transformation from theory to practice. Our concern is thus that even when information and knowledge are available, information is not used to its full potential.

A recommendation forwarded in several studies has been to focus on species conservation also outside protected areas [33]. Urban areas are no exception. The UN states, ‘Cities are rethinking urban space, not only from the perspective of health, but also ecology. They are recognizing the need to promote inclusive planning and to take regional dimensions into account’ [34]. The EU underlines how green infrastructure in urban areas “… plays a critical and increasingly important role in biodiversity conservation efforts.” [35]. Natural areas tend to be small and fragmented in urban settings, however, as has been documented in international [36] and Norwegian studies since the 1990s [37]. This does not imply they are of no value to biodiversity; however, due to their smaller size, they provide merely a limited range of niches and a limited number of habitats to a limited number of species. We hypothesize that applying principles developed within the framework of landscape ecology can help mitigate some of the negative effects of this.

The approach we describe originated from work related to a landscape architecture master thesis project [38], which has been further elaborated. Our research objective was to describe and test how landscape ecology can be integrated more explicitly with all stages of a planning and design process, as discussed by Hersperger, Grădinaru, Pierri Daunt, Imhof, and Fan [26]. In addition, we wanted to identify possible problems that, e.g., landscape architects and planners may face in such processes.

Specifically, our research questions were:

1. How can landscape ecological knowledge be integrated with a planning and design process?
2. Do planners and designers have the knowledge they need for a “landscape ecological approach”?

## 2. Method

### 2.1. Research Framework and Selection of Case Study

We wanted to use a case-study approach to examine whether theoretical principles of landscape ecology could be used by landscape architects in a typical planning situation. Our research approach consisted of nine steps:

- Define case study criteria;
- Identify case study municipality;
- Municipality data capture and analysis;
- Select landscape ecological principle(s);
- Crude regional analyses;
- Identify local study area for design;
- Categorize existing land-use/cover in the local study area;
- Define aims and design priorities and management regimes for the local study area;
- Design and visualize.

The steps are described in more detail below.

Our first step was to identify criteria for selecting a suitable case study area, and we agreed on the following:

- Data on natural conditions, species, nature types, etc., should be available in national databases and have been updated within the last 10 years;
- There should be diverse natural conditions and species assemblages;
- The area should be exposed to pressure from population and infrastructure development;
- The area should be within reach for a one-day visit.

**Figure 1:**
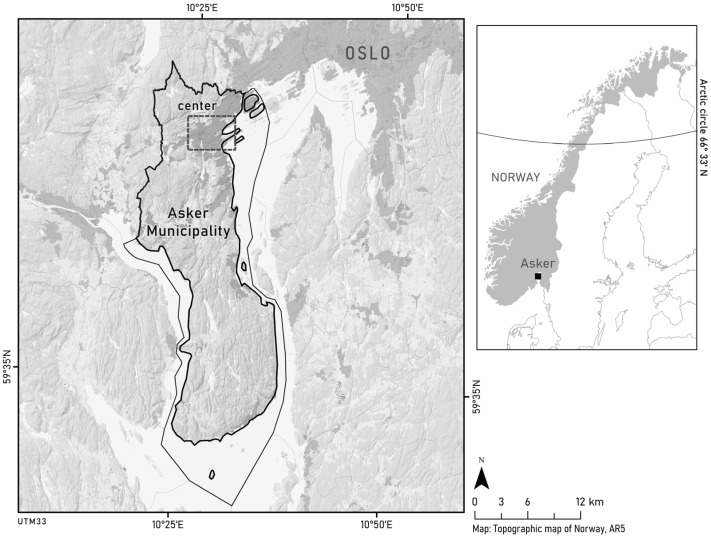
Asker municipality in south-eastern Norway. The solid lines in the left figure outline the municipality boundary (only land area and water included). The small square mark the center area of the municipality.

A lot of data in Norway is gathered on the scale of a municipality. In addition, the municipality is responsible for land-use planning. Thus, we decided to search for municipalities meeting the above criteria, and found that Asker municipality (Figure 1) met all of them.

Our next step was to gather various types of data on abiotic and biotic aspects of Asker municipality. Thus, we collected thematic maps on geology and soil, landform, and vegetation cover, occurrences of red-listed species and nature types, and built-up land. These maps are available free of charge in Norway. While our inspection could be interpreted to illuminate multiple challenges, it also clearly illustrated how fragmentation due to infrastructure and urban development and agriculture could represent challenges to the movement for most land-living animal species. Several studies have documented how connectivity is important to various species, ranging from butterfly and plant specialists [39] to grizzly bears [40] and fish [41]. Although there are situations where connectivity may be harmful [42], in general, recommendations have emphasized the need to ensure connectivity between fragmented habitat patches to enable movement, and thus ensure genetically sound populations and species persistence in the long term [39,43,44]. Having familiarized ourselves with the municipality through studying these maps, we decided to emphasize design that could help mitigate fragmentation effects and chose connectivity as the landscape ecology principle to work with in our design. While it is possible to calculate fragmentation and various additional aspects of landscape content and composition using tools and indices, such as Fragstats™ and GIS software [45], this is rarely an approach used by landscape architects and land-use planners in Norway [46], thus we decided not to include this type of measures.

**Figure 2:**
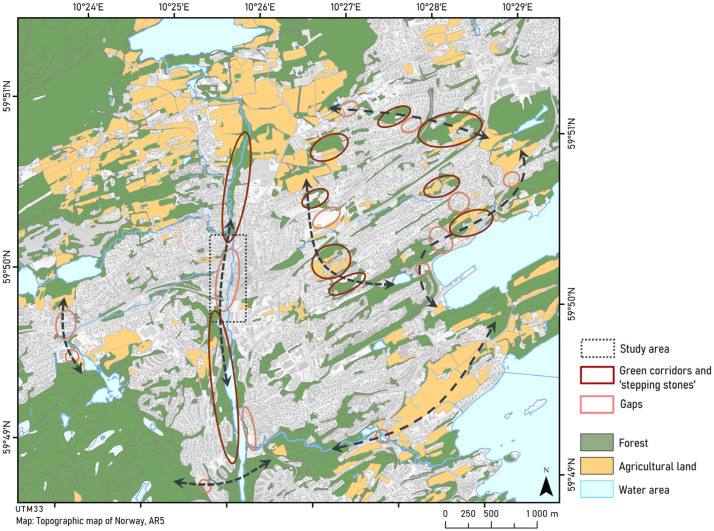
An illustration of the outcome of our visual inspection of the area in a more regional context. Arrows mark our assessment of potential movement routes for terrestrial species, and the “gap” we decided to focus on is outlined.

To make sure we were seeing the potential study area in the context of the surrounding landscape, we also changed our scale and zoomed out. It was outside the scope of this study to conduct regional landscape analysis, as will also be the case in most real-life planning situations. Nevertheless, wanting to make sure our plan would connect to the surrounding landscape, we decided to conduct a crude visual inspection of the readily available regional land cover and land-use maps. We identified areas of forest, water, and agriculture distributed within and around the built-up land. By a visual inspection of the maps, we identified where there were apparent gaps between smaller patches of non-developed land. By doing this, we clearly saw how our case study area had the potential to fill one of these gaps. Our analysis is illustrated in Figure 2.

**Figure 3:**
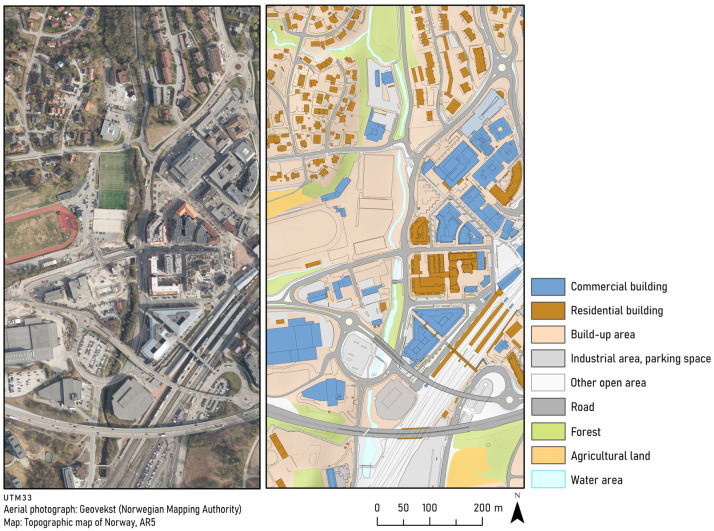
Our case study area with the river corridor in a densely built-up landscape.

To identify an area suitable for a more detailed design approach focused on connectivity, we looked for a gap with an existing natural linear element, and we identified the river corridor as a potential site (Figure 3).

Zooming in on our chosen case study area, we identified all land-use/cover types present. As the land-use/cover typology used for our land cover map was both too much and less suitable for our purpose, we decided we needed to develop a simple typology based on the assessment of human influence on the land. Therefore, we categorized existing land-use into four classes: mainly natural, natural, human, and mainly human. Then, we defined as our design strategy to aim for connecting, to the extent possible, patches of mainly natural land cover along the river, while also acknowledging the great potential for the river corridor for recreation and nature experience for the local population.

### 2.2. Case Study: Asker Municipality and Asker River Corridor

Asker municipality is located just outside Oslo in south-eastern Norway (Figure 1). Asker municipality covers 189 km^2^, and has a population of ca. 60,000 inhabitants. The population density is 259 people per square km (accessed on 1 January 2022, www.ssb.no), compared to a national average of 15. Major infrastructure, in terms of roads and railways, traverse the municipality.

Land cover in Asker is heterogeneous. The region is biologically rich, with a wide range of habitat types, including both ocean coastline, hilly forested areas, and calcareous rocks. Calcareous rocks are uncommon in Norway, where acidic bedrock poor in nutrients often dominates. Asker has a considerable population of oak trees (*Quercus* spp.) which the Norwegian Environment Agency has designated as a nature type of particular interest [47]. At the coastal edge of the municipality, there are also a significant proportion of protected calcareous lime forests.

Asker center, the focus area of this study, is a densely populated urbanized area with a heterogeneous land cover, divided by the river Askerelva (Figure 3). The river and surrounding areas contribute to the municipality’s heterogeneity, as do the recreational areas, transport infrastructure, and the commercial center. In addition, there are also elements of parks and gardens that may contribute habitat and ecological functions in this small urban center. Several data sources with biological and land-use/land cover information are freely available in Norway. The most important ones in this context were data on land-use (Land-use/cover map (scale 1:5000)) [48] and Orthophotos [48]. We also used data from ‘Naturbase’, containing data on species observations, protected areas, valuable nature types, and recreational areas available from the Norwegian Environment Agency [49], and we collected data on the presence of red-listed species and nature types present from the Norwegian Biodiversity Information Centre [50]. This was complemented by the municipality of Asker’s survey of biological diversity [51]. In addition, we used planning documents from the municipality and private stakeholders.

The process builds on the familiar landscape planning principles of Ian McHarg [52] using thematic maps and overlay techniques. Specifically, we used the thematic maps, which showed bedrock, soils, landform, vegetation cover, nature types and protected areas, particularly valued nature types, recordings of red-listed species, habitat areas, and built-up land.

## 3. Results

We found that applying a four-stage process, only slightly revised from the one described by Hersperger, Grădinaru, Pierri Daunt, Imhof, and Fan [26] worked well. In the first stage, knowledge of land-use/cover and characteristics of the study area was important. This introduced us to many biotic and abiotic aspects of the municipal landscape, areas of potentially conflicting interests, and areas with important features, for example, valued nature types and areas with a high density of red-listed species.

Having decided to emphasize connectivity as our landscape ecological principle, we used both a regional and a local map to identify features that contributed to connecting blue-green elements/patches and bridging potential barriers between them, i.e., the potential elements in the blue-green structure or the ‘emerald necklace’ (see emeraldnecklace.org). We found we were able to identify existing and missing links, and land that could function as possible barriers (hinder movement) and corridor (facilitate movement) structures. Thus, our result was that even our merely visual inspection of regional land-use/land cover maps gave us a good overview of the landscape, and in particular key elements fragmenting the remaining more natural sites, i.e., forested and agricultural land, and rivers and lakes. We identified some larger areas of non-developed land, which potentially connect municipal blue-green systems with those at regional levels. From a landscape ecological point of view, these larger non-developed areas are particularly important to map as they provide habitat for species requiring larger habitats or habitats with less influence from the surrounding landscape, and important in our context, provide habitat for some species, which may be able to recolonize new habitat if connected.

When looking for areas that could function as elements in a blue–green structure at the local scale, if subjected to a landscape ecology-based design, we realized that we wanted to keep our analyses on a more general level. Therefore, we did not base the process on any scientific analyses of, for example, resistance of the land cover types to movement by particular species or indicator species. If we had aimed at this level of detail, we would have needed to identify particular species or species groups to investigate and assess their mobility. In our experience, this is rarely done in a typical Norwegian planning situation. Based on our test, we believe an approach that can be applied is to use the available land cover/land-use map (scale 1:5000), accompanied by orthophotos (true color, scale 1:15,000), to provide information about areas of importance from a biodiversity perspective (available in national databases), and plans for future development. We found that locating species observations and nature types on the study site map revealed several important locations. This also provided us with potential ‘bottleneck areas’, i.e., where housing and infrastructure appeared to be about to cut off connectivity between natural areas. We could also identify zones of potential interaction, e.g., boundaries between housing areas and natural areas. The land-use map illustrates how narrow the corridor is, and how close future housing and commercial developments and large infrastructure projects are. We found this to be useful input into our design of the river corridor.

Along the river corridor, we assessed the different land-use/land cover elements present. We found that the existing land-use/land cover classification was not ideal for our purpose, for example, elements were mapped as built-up when it also contained smaller elements with grass and trees. In addition, we wanted a typology that was in line with our design ideas and the main factor we believed to be influencing the perceived structural connectivity of more natural areas, which was the human presence. Thus, we assigned all land to one of four categories of land cover/land-use representing more natural (e.g., forests, wetlands, and lakes), or more human-dominated (e.g., residential and industrial) types. This crude categorization worked as a typology to help us get an overview of where there was human-dominated land, potentially affecting the structural connectivity of the more natural areas. The process also revealed locations where the more human-dominated types of land-use, e.g., residential or commercial, created a gap in the connectedness of more natural types, such as small forests. Based on our aim to increase structural connectivity, we found that it would be useful to focus on four types of land-use elements along the river corridor: private gardens, lawns, road verges, and waterways. These are landscape elements that appear to match the description by Forman [36], being smaller elements that occur repeatedly within the study area and, thus, are suitable candidates for change by design. However, we realized that it would not be beneficial to apply the same design to all elements of the same type, for example, convert all lawns to mainly natural woodland. This would easily come in conflict with other potential uses of the area, e.g., for recreation. Thus, we found that our design suggestions had to be based on the specific location of each area, and its relation to neighboring areas.

In the more detailed design stage, we wanted to integrate data on vulnerable species and valued nature types in as much detail as possible, by using data from the national biodiversity database “Artsdatabanken”. This was intended to function as a biodiversity knowledge base for the design, and to help us develop principles for design with emphasis on vegetation structure and selection of species, typical tasks within the framework of landscape architecture and land-use planning. However, this turned out to be a difficult process. In total, 555 species have been recorded in Asker municipality. The Norwegian Red List classifies species as belonging to one of four categories: critically threatened (CR), highly threatened (EN), near threatened (NT), or vulnerable (VU) [53]. In Asker, 4.646 single observations belonged to one of these categories. However, more than a thousand of observations were recorded before 1970. It was also unclear to what extent the quality of the data was ensured and by whom, and the spatial accuracy was variable. Moreover, only to a small extent had the material been systematized and adapted to use for planning purposes. Still, there were several vulnerable species in the area, primarily in and along the river corridor (mussels, birds, and insect species). In addition, we identified nature types subjected to protective guidelines according to the Nature Diversity Act [54]. These included hollow oaks (*Quercus rubra*) and rich deciduous woodland. Overall, though, the biodiversity data were overwhelming in volume and detail, and we found it very difficult to know how to make the best use of it.

We decided to base our vegetation models on a morphological approach by classifying vegetation according to the variation in the composition of layers inspired by Gustavsson [55]. Gustavsson developed a novel classification that could be used to conduct empirical studies of forest landscape change, and a tool in landscape planning and design [55]. His focus was more on the tree layer’s morphology, an approach seemingly suitable also for our purpose. By using Gustavsson’s approach to creating vegetation layers, we wanted to identify plant species that would strengthen the function of the different area categories. For instance, in the ‘mainly natural areas’ category, it was important to use species that would provide a heterogeneous horizontal and vertical structure, as this would ensure a larger diversity of species and provide additional habitat. Multiple vertical layers of vegetation would, for example, provide better cover for ground-moving species, species that prefer to move with a low risk of being detected, e.g., badgers or roe deer. In addition, a heterogeneous horizontal structure would allow more species to find needed resources simply by adding variability in space and time, e.g., a wider range of plant species and species flowering or producing fruit at different times during the season. Furthermore, the chances of succeeding in creating a habitat for a diverse number of species are higher if we give priority to plant species that are beneficial to other species, for example, plants that provide nectar and pollen to insects, or trees suitable for cavity-nesting birds. As a result, vegetation became a tool in both separating and linking the different areas, their uses, and functions. To identify possible species, we decided to look at four criteria; (i) whether they provided resources for other species, (ii) their ecological characteristics (e.g., vulnerability, population status, suitability to the area), (iii) considerations of people (e.g., toxic, smelling, and allergenic), (iv) specific requirements (pH, humidity, and local climate). We found that this worked well.

In our final stage in the process, we brought the different types of information together, aiming to test how one can apply landscape ecological knowledge in a detailed design to meet a more specific objective (e.g., increasing connectivity to benefit biodiversity) in an area. We used our typology and the information about the spatial distribution of the elements described above and found that this approach worked as intended. For example, there was a large total area of lawn surrounding the existing buildings and along the roads, where we suggested developing a less managed and human-influenced land-use type. Assessing the entire river corridor, instead of the “one property at a time” approach, we believe we managed to create a design that met our aim, to increase the structural connectivity of the less human-influenced land-use/land cover along the river corridor. However, we did also keep certain areas as more “cultivated”, i.e., in the “mainly human” category.

**Figure 4:**
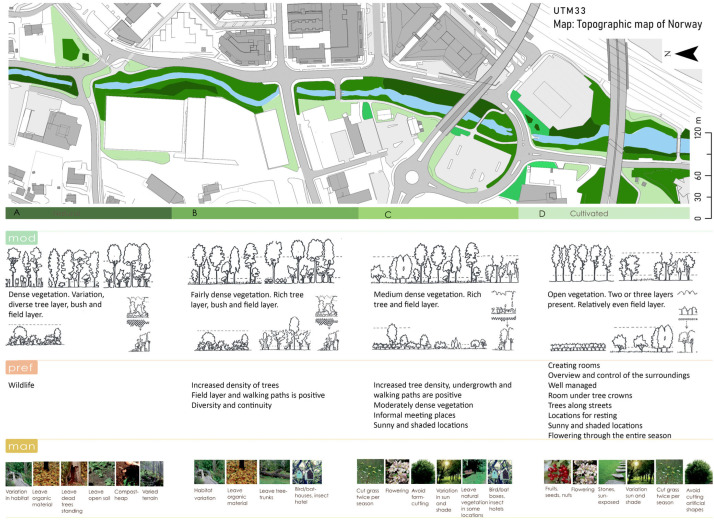
An illustration providing an overview of the distribution of the four categories of our typology along the river corridor. Below the map are some ideas regarding the design, and the preferences. At the bottom are images intended as illustrations of management guidelines.

**Figure 5:**
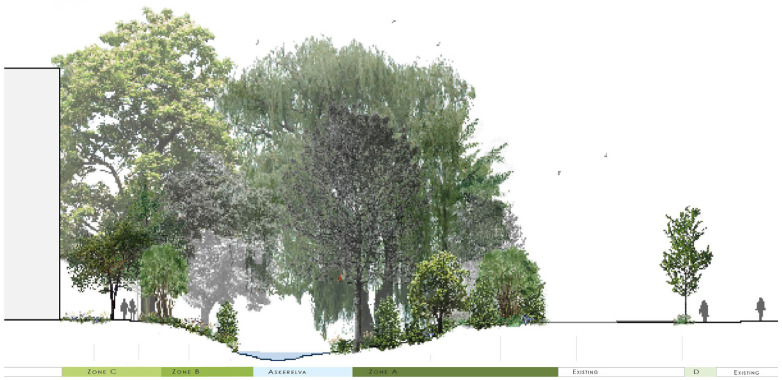
Example of illustrations. Colors illustrate categories according to the typology.

Finally, we realized we needed to include information about our design ideas and management advice for our different categories. This is illustrated in Figure 4. We also made illustrations to visualize our design, such as the one presented in Figure 5.

## 4. Discussion

Hersperger, Grădinaru, Pierri Daunt, Imhof, and Fan [26] documented how landscape ecological concepts are often used in the analysis of a study area, but less frequently integrated with the other steps of the planning process. Furthermore, they concluded that a clear link from the concepts to planning remained an exception [26]. We aimed to establish such as link, and to test how landscape ecology could contribute to the entire planning and design process, and improved integration of ecological data and knowledge about ecological processes in the landscape.

In our test case, we used landscape ecology in the first stage, goal establishment [26], as we wanted to focus on connectivity. To ensure connectivity in an urban or peri-urban landscape, a potential planning approach includes the establishment of a blue-green network, also sometimes called an “emerald necklace”. Typically, this necklace consists of various blue and green elements (e.g., rivers, parks, ponds, urban woodlands, and even brownfields), and different types of ‘links’ between these [36,56], i.e., patches and corridors using a landscape ecology terminology [10]. The concept of an “emerald necklace” is well-established within landscape architecture and landscape planning, and thus worked as a unifying concept between the disciplines. This concept also provided a useful base for discussing how both elements and links should have characteristics that may influence and enhance their function, e.g., as a habitat for species of interest, or as corridors for their movement [36]. Ensuring the connectedness of the network through these links or the land-use matrix itself is important from an ecological perspective [11,57]. From our experience, we consider finding a useful unifying concept or idea to have potential when aiming to make ecological knowledge better integrated into a planning and design process. Furthermore, we found it useful to place the design area in a larger landscape ecological context through a crude regional assessment even though it was based only on a visual map inspection. We are concerned this regional perspective often may be given a low priority in many planning processes. This is probably due to the commonly tight economic and time constraints in municipal planning, where all emphasis is directed to a clearly outlined designated planning area [36].

It was not possible within the scope of this project to do a field-based mapping of species diversity. This was neither an aim nor is it likely to be so in real planning situations. Rather, we aimed to use available information to capture as much relevant biological information as possible, and to do a crude assessment on the spatial pattern. Our experience from this rather restricted test is in line with several other studies, such as the one from Stockholm, and from Thorén and Saglie’s study from Oslo [32,58]. The task immediately became overwhelming. Most professional practitioners will not have the training required to assess and analyze large amounts of ecological or species data, or to judge the importance of managing an area for one species over the other. Planning processes are usually speedy, and biodiversity is an issue that may be omitted or randomly treated.

While vegetation, land cover and land-use maps may provide useful information in a planning situation, it is not necessarily information that is easy to apply directly in a design process. Our four categories of desired land-use intensity, where we assigned each land parcel to one of these to operationalize this background information for further planning, worked well in our context. As connectivity was in focus at the landscape scale, it seemed appropriate to aim for a continuous ‘mainly natural’ category, under the assumption that ‘mainly natural’ would maximize that function for the largest number of species. In general, we found this a very useful simplification of the original land-use/cover typology that also reduced the need for specialized ecological knowledge.

In contrast to maps of existing land-use/land cover, these categories also represent a prioritization of objectives for each parcel of land, not only a description of the current state. The prioritization was based on the importance to safeguard biodiversity and planning for human needs. The main considerations in this process were analyses of biodiversity in the river corridor, in terms of records of red-listed and other species and vulnerable nature types, and assumptions concerning needs related to recreation.

We aimed to focus on multifunctionality [59] in the study areas, while at the same time spatially separating the least compatible functions. To achieve this, we also developed a set of guidelines for each category. For instance, category A (‘mainly natural’ areas) would have a total ban on introduced species, dead trees were to be left alone, and heterogeneity of vegetation and habitat was a priority. In contrast, in the ‘mainly human’ category (D), we allowed exotic species, and we gave aesthetics and accessibility priority. Figure 4 gives an overview of the distribution of the categories along the river corridor. To use the different categories along the gradient from ‘mainly natural’ areas to ‘mainly human’ areas, while keeping the landscape ecological perspective, it was necessary to work both at a local scale and a larger scale. This implied balancing the occurrence and distribution of the different spatial categories.

From our perspective, the process we described and tested with the stages, including assessment, selection, and application of relevant and applicable principles prior to design and decision-making, functioned as intended. It did contribute to a useful work trajectory, ensuring that important perspectives were included at the right stage in the process. It also demonstrated that using principles and ideas based on landscape ecology could guide a planning and design process on multiple spatial scales from the wider landscape to the local analyses. This is not to say there is no room for improvement. For instance, deciding on the best range of scales to include is something that needs further testing and refinement. This is also a theme that should be further elaborated in a next step which would be testing the approach described in a real-world planning situation. A real-world situation would also involve various stakeholders and a gathering of feedback and experiences from these. In addition, monitoring the functioning of the area over time would be important, as input in a potential later refinement of the design.

In a real planning situation, managing biodiversity and blue–green structures in urban areas is the result of management on a range of scales, and by an array of institutions. This includes the individual gardeners’ day-to-day urban park management, and the more general regional and national regulations and laws. According to Ernstson [30], there is little awareness of the fact that consideration on a range of scales is necessary for ensuring the ecological functions of green areas. This implies that there is a need for more detailed and general strategies. Löfvenhaft has demonstrated how this can be approached, with the National Urban Park in Stockholm as an example [60]. There, the initial step was to develop a classification system to map the urban vegetation both on public and private land and to use the information to manage selected indicator species. This would allow for a detailed and targeted approach to hopefully strengthen the populations of these species. However, a complicating factor is the complexity involved, as well as the frequent lack of transdisciplinarity [61], which could hinder the success of the approach.

Working transdisciplinary may be exactly what is needed in future urban planning though, as was demonstrated in Oslo [62]. In particular, we hope to see a closer collaboration between biologists, landscape ecologists, landscape architects, architects, and planners in the future. This is in line with what was also outlined by Hersperger, Grădinaru, Pierri Daunt, Imhof, and Fan [26] calling for more dialogue and Trammell, Carter, Haby, and Taylor [27] suggesting to have more landscape ecologists present in large agencies involved in landscape planning and management. Based on our experience, we would argue that to manage an area in the best way from a biodiversity perspective, ecological data should be interpreted by an ecologist. This would contribute to ascertaining how to assign priorities, when necessary, the validity of the data (for example based on the number, observer, and age of observations), and what are the relative importance (for example based on rarity). We thus consider there to be an urgent need for ecologists to contribute to the information flow in planning processes and the transdisciplinary process needed to face the current challenges.

In general, our answer to our second research question is indeed that we suspect that neither landscape architects and planners nor ecologists alone have the knowledge needed for a “landscape ecological approach” to planning. In a process of changing land-use, the amount of input information and the decision-making involved is demanding and complex. Therefore, we fully support the suggestion by Trammell, Carter, Haby, and Taylor [27], and believe this to be the best way forward.

It is rather obvious that when we interfere with and change a landscape, some species may benefit from the planned changes, while others are likely to suffer. To ensure the best possible outcome from an ecological perspective, it is important to have clearly defined aims. The result is likely to be different if you plan for maximizing species diversity in the area, rather than aiming to protect a particular species. In our study, we decided to emphasize improved connectivity as an overall strategy to improve the habitats for a range of species. We believe multiple species will benefit from our resulting plan for the river corridor as it defines different categories of land-use and strengthens their individual spatial connectedness. This is based on the well-documented positive effects of improved connectivity when habitats are fragmented [44], as is the case in our study area.

Based on this study, we suggest that the design process needs to be broadened, to better integrate knowledge from, e.g., ecological disciplines into land-use planning and design. We did not specifically consider Nature-based solution (see, e.g., [63]), but this is also something that we anticipate could be a useful future approach for ensuring cross-disciplinarity [64]. For this to happen, available knowledge generated in biology or ecology must be translated into something more applicable in planning and design, in line with the points made by Carter, Pilliod, Haby, Prentice, Aldridge, Anderson, Bowen, Bradford, Cushman, DeVivo, Duniway, Hathaway, Nelson, Schultz, Schuster, Trammell, and Weltzin [17]. Here, in particular, landscape ecology can be a tool, but landscape architects and planners may also need to broaden their knowledge of biology and ecological processes. If we are to meet the challenge of protecting biodiversity, cross-disciplinarity must be moved from rhetoric to reality. We also recommend always swapping between different spatial scales and bringing landscape ecology thinking into the process on all relevant scales.

Finally, design based on ecological principles may be considered unattractive and even un-aesthetic [65]. Thus, the visualization of design proposals is an important part of the project to show that such principles can indeed help create beautiful and diverse outdoor environments. This is again a task where cross-disciplinarity can be helpful, as this is typically a task for which designers and landscape architects are well-trained.

## 5. Conclusions

Landscape ecology has a lot to contribute ensuring better integration of ecology in land-use planning and design, and can aid in multiple stages of the processes. With the land, allocation of land to different uses and spatial patterns as common denominators, landscape architects, land-use planners, and landscape ecologists should have a solid foundation for collaboration. In this project, we decided to emphasize a major challenge in this case study landscape, connectivity. This is not to say that we did not also keep other principles, e.g., heterogeneity, in mind. In another landscape type, the situation may be different, and, e.g., establishment of new patches may be the key objective. However, it is important to be aware that these principles are not simply applicable in their “existing format”. Rather, there is an adaptation process required where theory needs to be matched with local situations and relevant data gathered and applied. For landscape ecology to achieve a wider application, it may be that landscape ecologists should focus on how to ease this process.

A major finding from our study is that the step from landscape ecological theory to practical application is challenging. A range of factors influences this. Among the more important in our perspective is the lack of municipal or regional landscape analysis based on landscape ecological principles. Such an analysis could be used to prioritize landscape elements important to a blue–green infrastructure on a wider spatial scale, and to identify areas that could, or should, be given priority as addition to this. Furthermore, much biodiversity information, e.g., species occurrences, is not available in a way that makes it useful for professional practitioners.

Another recommendation is to increase the degree of participation in each stage of the planning and design process. We anticipate that planning and design with stronger integration of ecological considerations, may both require trade-offs (e.g., between species habitat and aesthetics) and lead to novel visual expressions. We believe that wide participation can ensure acceptance of the potentially involved tradeoffs and end results.

The approach described in this paper is not that different from what is commonly used in landscape architecture. However, what is novel in our approach is the way landscape ecology and ecological knowledge is integrated into every step in the planning and design process and on all spatial scales, from regional assessments to the selection of plant species on a detailed design level. We hope that this can be an example of an approach to be further developed, adapted, and applied in other projects, and act as inspiration for further collaboration between landscape ecologists, landscape architects, and land-use planners, as we believe this could be a way forward.

## Data Availability

Not applicable.
